# Demography of the Early Neolithic Population in Central Balkans: Population Dynamics Reconstruction Using Summed Radiocarbon Probability Distributions

**DOI:** 10.1371/journal.pone.0160832

**Published:** 2016-08-10

**Authors:** Marko Porčić, Tamara Blagojević, Sofija Stefanović

**Affiliations:** Laboratory for Bioarchaeology, Department of Archaeology, Faculty of Philosophy, University of Belgrade, Belgrade, Serbia; University of Florence, ITALY

## Abstract

The Central Balkans region is of great importance for understanding the spread of the Neolithic in Europe but the Early Neolithic population dynamics of the region is unknown. In this study we apply the method of summed calibrated probability distributions to a set of published radiocarbon dates from the Republic of Serbia in order to reconstruct population dynamics in the Early Neolithic in this part of the Central Balkans. The results indicate that there was a significant population growth after ~6200 calBC, when the Neolithic was introduced into the region, followed by a bust at the end of the Early Neolithic phase (~5400 calBC). These results are broadly consistent with the predictions of the Neolithic Demographic Transition theory and the patterns of population booms and busts detected in other regions of Europe. These results suggest that the cultural process that underlies the patterns observed in Central and Western Europe was also in operation in the Central Balkan Neolithic and that the population increase component of this process can be considered as an important factor for the spread of the Neolithic as envisioned in the demic diffusion hypothesis.

## Introduction

It is widely accepted that the Neolithic way of life was introduced to Europe from the Near East, with Anatolia being the most important "source" region [1, 2]. There is little doubt that the spread of the Neolithic involved the actual migration of people from the Near East to Europe as demonstrated by accumulated genetic [3–7] and bioarchaeological evidence [8, 9]. The results of the mathematical modeling have shown that the demic diffusion (migration) was the main mechanism of spread [10, 11], although the process was by no means uniform. After the initial Neolithic colonization in Greece in the first half of the 7th millennium calBC [12], the Neolithic spread to the rest of the Europe via two major routes: the maritime Mediterranean route (Greece –eastern and western Adriatic coast—southern France—Iberia) and the continental route (Greece—Balkans—Central Europe—Western and Eastern Europe).

The Central Balkans area was one of the main corridors for the spread of the Neolithic from Greece further into Central Europe and beyond. The appearance of the first Neolithic in Central Balkans is related to the Starčevo culture, which is a part of the wider Early Neolithic cultural Starčevo-Körös-Criş complex [13, 14]. The beginning of the Starčevo culture is conventionally dated to 6200 calBC as suggested by the earliest dates from the sites of Blagotin, Donja Branjevina and the first appearance of Starčevo pottery in Mesolithic contexts in the Danube Gorges [15–17]. The end of Starčevo culture and the Early Neolithic period in Central Balkans is dated to ~5300 calBC which coincides with the appearance of the Late Neolithic Vinča culture (5300–4500 calBC) with a markedly different cultural repertoire: pottery style and technology, architecture, settlement organization and copper metallurgy [13, 14, 18–22].

Given the central role of this region for the spread of the Neolithic to Europe, the archaeological reconstruction of demography of the Central Balkan Neolithic societies is necessary as demographic aspects have a prominent role in almost all theories, models and hypotheses proposed to explain the phenomenon. In addition, knowledge of demography is needed in order to understand socio-cultural processes, especially changes associated with the shift from Late Neolithic to Early Neolithic in Central Balkans, archaeologically documented as changes in technology, settlement, architecture, subsistence, and stylistic features of material culture [18, 20–25]. Despite the fact that the Early Neolithic in Central Balkans has been intensively studied during the past 80 years little is known about the demography of the Early Neolithic communities. A study undertaken by Whittle et al. [15] had demographic implications but was primarily concerned with establishing the absolute chronology of the spread of the Neolithic. The authors suggested a model of gradual beginning before and around 6000 BC, with the spread of new ideas from south to north, and communities being scattered at the sides of the Danube and its major tributaries. The later phase of Early Neolithic dispersal is characterized by much more sites throughout the region. Whittle et al. did not find Ammerman and Cavalli-Sfoza’s “wave of advance” model [26] to be appropriate in explaining the spread of Neolithic at this territory [15]. They proposed a scenario of limited colonization and indigenous acculturation and adjustment to a regional scale, noting that this model is difficult to prove with traces of indigenous populations missing from the record, except for the region of the Danube Gorges [15].

Paleodemographic studies of the Mesolithic-Neolithic transition in Central Balkans have been limited to the Danube Gorges [27–31], but the cultural process in this region is specific and is not representative of the situation in the rest of the Central Balkans. The Danube Gorges population represents a special case, considering that hunter-gatherers in that area, who accepted some of the elements of Neolithic package, were confined to the Danube Gorges region [16, 32–34], and their population dynamics cannot be extrapolated to the rest of the Central Balkans where almost no Mesolithic populations were detected [35].

What pattern should we expect to find? The main demographic process in the Neolithic was the process of the Neolithic Demographic Transition (NDT) [36]. The theory of the NDT as formulated by Bocquet-Appel suggests that the NDT was a two stage process [37–42]. In the first stage the population increased exponentially with high intrinsic growth rates (between 1% and 2%). This growth was caused by the growth in fertility (the number of children) enabled by a diet rich in high energy carbohydrates coming from cereals and sedentary lifestyle. This increase in fertility in Neolithic communities was soon followed by an increase in mortality. Increased mortality, especially among infants, is a result of numerous factors—introduction of new pathogens, contamination by feces, reduced breastfeeding, malnourishment resulting from the less diverse diet poor in protein and essential nutrients, and higher workload of the Neolithic people [36, 37]. Therefore, in the second stage of the NDT mortality (death rate) caught up with fertility (birth rate) and the population growth stopped. The strong support for the NDT theory has been found worldwide from both skeletal and settlement data [42–49]. A recent study by Shennan et al. [50] and Timpson et al. [51] using summed calibrated radiocarbon probability distributions method demonstrated convincing patterns of the NDT in various regions of Western, Northern and Central Europe. The pattern detected by these researchers consists of a population boom coinciding with the introduction of the Neolithic way of life followed by a bust few centuries later. Based on these theoretical and empirical results we should expect to find these patterns of boom and bust in the Early Neolithic of Central Balkans as well.

The aim of the research presented in this paper is the reconstruction of population dynamics in the Early Neolithic of Central Balkans using summed calibrated radiocarbon probability distributions (SCPD) as population proxy. Demography plays a central role in anthropological theories of socio-cultural evolution, adaptation of human societies and cultural transmission (e.g. [52, 53–58]). Given its theoretical importance, archaeologists have been developing methods to extract demographic information about past populations from various kinds of archaeological evidence [59–61]. These methods have often been criticized as biased and imprecise (e.g. [62, 63–66]), but great efforts have been invested over the years in refining and upgrading in order to resolve problems raised by critics. Even though paleodemographic proxies and methods may be less reliable when looked at individually, taken together they may provide robust conclusions when the results of different methods converge, or open new questions when there is divergence in results.

Given the lack of information about the demographic dynamics in Central Balkans and the importance of the region for understanding the spread of the Neolithic into Europe, this study is long overdue and our intention is to begin to fill the gap in our knowledge about the demographic aspects of the Balkan Neolithic. We stress that this is the first application of the SCPD method to reconstruct population dynamics of the Neolithic people in Central Balkans, who were the bearers of the Starčevo and Vinča cultures.

## Data and Method

**Fig 1:**
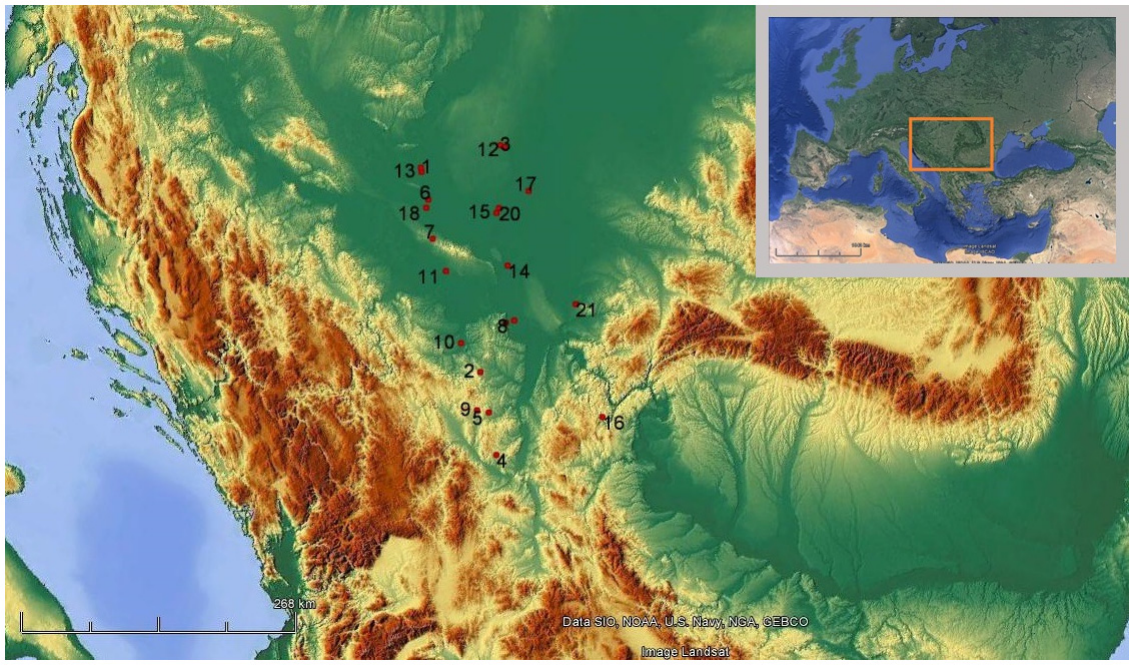
Early Neolithic sites with radiocarbon dates included in the study. 1.Apatin; 2.Banja-Aranđelovac; 3.Biserna Obala Nosa; 4.Blagotin-Poljna; 5.Divostin I; 6.Donja Branjevina; 7.Golokut-Vizić; 8.Grad Starčevo; 9.Grivac; 10.Jaričište; 11.Kudoš-Šašinci; 12.Ludoš-Budžak; 13.Magareći Mlin; 14.Perlez-Batka “C”; 15.Ribnjak-Bečej; 16.Rudna Glava; 17.Sajan-Domboš; 18.Topole-Bač; 19.Vinča-Belo Brdo; 20.Vinogradi-Bečej; 21.Vršac-At (map produced by Tamara Blagojević using Google Earth, on a map layer downloaded from http://maps-for-free.com/, accessed on 20th May 2016).

This study included published radiocarbon dates from the Early Neolithic sites from the Republic of Serbia (Fig 1). Dates from the Danube Gorges were not included in the analysis for the reasons stated in the Introduction. Moreover, the inclusion of these dates would create a strong research bias, as there are over 300 Mesolithic and Early Neolithic dates from the Danube Gorges alone compared to the total of 72 dates coming from the rest of Serbia.

Measures of chronometric hygiene were undertaken meaning that radiocarbon dates with large standard errors (greater than 180 radiocarbon years) were removed from the database. We excluded 15 dates—13 from Grivac site, where standard errors were from 425 up to 825 radiocarbon years; and two dates from site Lepeničko polje near Kragujevac, with standard errors of 450 and 490 radiocarbon years. It should also be noted that all dated samples were pottery fragments, not suitable for C14 dating in the first place. Also, some of the dates were omitted as they seemed to be suspiciously old when individually calibrated. These are the dates from Starčevo-Grad (GrN-6628) and Blagotin (OxA-8608), both substantially older than 6200 calBC which is currently accepted upper limit for the beginning of the Neolithic in this region. After these exclusions, there were 72 Early Neolithic dates left, from 21 Starčevo sites. The list of the Early Neolithic dates used in the analysis is presented in the electronic supplement (S1 Database, Starčevo sites). The sample size is admittedly small which implies low statistical power of the method (the ability to detect statistical deviations from the null model), but this fact should make us even more confident in the patterns which cross the threshold of statistical significance.

In order to reconstruct population dynamics we applied the summed calibrated radiocarbon probability distribution (SCPD) method. This method was originally introduced by J. Rick [67], but in this paper we apply the extended SPCD method as developed by Shennan et al. [50] and Timpson et al. [51] which accounts for research bias, effects of taphonomy and calibration curve on the summed probabilities and provides an explicit test for the statistical significance of the demographic signal. The method was implemented in R [68] with *Bchron* package used for the calibration of radiocarbon dates [69]. The validity of the summed radiocarbon probability method used in this paper has also been hotly debated [64–66, 70–73], however recent improvements of the method successfully deal with most of the problems raised by the critics [50, 51, 74]. We present the summary of the Shennan-Timpson method in the paragraphs below.

The research bias results from the fact that radiocarbon samples are not collected randomly between and within sites and site-phases. The collection of radiocarbon dates is always driven by specific research interests and consequently the number of dates coming from different site-phases may often be a consequence of the researcher being more interested in one site-phase than another. In order to reduce this bias the binning procedure of radiocarbon dates within sites or site-phases was performed first. Radiocarbon dates are first binned into site-phases and then sorted in decreasing order within each site-phase. The dates within a given site-phase were further subdivided into bins if the difference between two adjacent dates was greater 200 radiocarbon years. The dates are first calibrated and summed within bins, with a bin sum normalized to the area of 1, and the resulting bin sums are then summed (between bins sum) and normalized to produce the final SCPD curve. This procedure controls for research bias when it comes to the frequency of samples per site-phase but it does not control for the bias stemming from the selection of sites from which to take samples. The binning procedure performed on the 72 Early Neolithic dates from this study produced 21 bins.

It is a well established fact that there is a loss of information about past events as an exponential function of their age. The implication is that we should expect to find less material (e.g. fewer samples to be dated) from greater time depths for taphonomic reasons alone. In order to address this source of bias, the taphonomic exponential curve equation developed by Surovell et al. [75] based on the terrestrial record of volcanic activity was used as a null model against which the statistical deviations of the empirical SCPD curve were assessed. This null model assumes that the underlying population was stationary (i.e. of uniform size through the time interval of interest) and that the taphonomy is the only factor, apart from the shape of the calibration curve, which affects the shape of the empirical SCPD curve. This means that even if the underlying population size was uniform through time (stationary population) we would expect the frequency of material remains (and by implication frequency of dates) to decrease exponentially as we go deeper into the past, and we would expect that to be reflected in the shape of the SCPD curve. In this step we deviate from the original formulation of the Shennan-Timpson method where the null model is constructed by fitting the exponential model to the empirical SCPD with an aim to account both for taphonomy and a secular population growth trend. This procedure is highly conservative because the fitted null model may account for both taphonomy and population trends as there is no way of telling whether an increase of probability density curve in time is due to taphonomy, population growth, or both. We make no assumptions about the secular population trend and use the null model curve which is completely independent of the data and which should model the effects of taphonomy alone.

In order to assess the statistical significance of the empirical SCPD pattern, a large number of simulated radiocarbon datasets is generated by randomly sampling calendar dates from the specified time interval according to the probabilities given by the null model. The number of dates for each simulated dataset is equal to the number of bins in the empirical dataset. This procedure is repeated many times resulting in a collection of simulated SCPDs generated by the null model. For the Early Neolithic dates in this study, we simulated 2000 null model SCPDs in the time interval between 6250 cal BC and 5250 calBC.

The sampled calendar dates are then "uncalibrated" by simulating a radiocarbon date which might have produced that particular calendar date given the laboratory measurement error value. For each simulated radiocarbon measurement an error value was assigned by sampling with replacement from the set of empirical radiocarbon standard error values. The "uncalibrated" dates were then recalibrated and summed to produce the simulated SCPD pattern.

In order to assess the statistical significance of the empirical SCPD pattern, the empirical SCPD curve was compared to the 95% confidence intervals calculated from the simulated SCPD values for each year of the time interval of interest. When the empirical SCPD is above or below the 95% confidence intervals, there is a statistically significant growth or decline of population relative to the null model. In order to control for false positives, as we would expect simulated SCPDs to be outside the 95% confidence intervals 5% of the time, a global significance statistic is calculated by transforming both empirical and simulated probability density values into Z scores, in relation to the simulated distribution for each time unit. Z scores which are outside the 95% confidence intervals are then summed both for the empirical and simulated curves. The empirical sum of Z scores is compared to the distribution of summed Z scores from simulated datasets. The global significance value is the relative frequency of simulated Z score sums which are equal to or greater than the empirical value.

## Results

**Fig 2:**
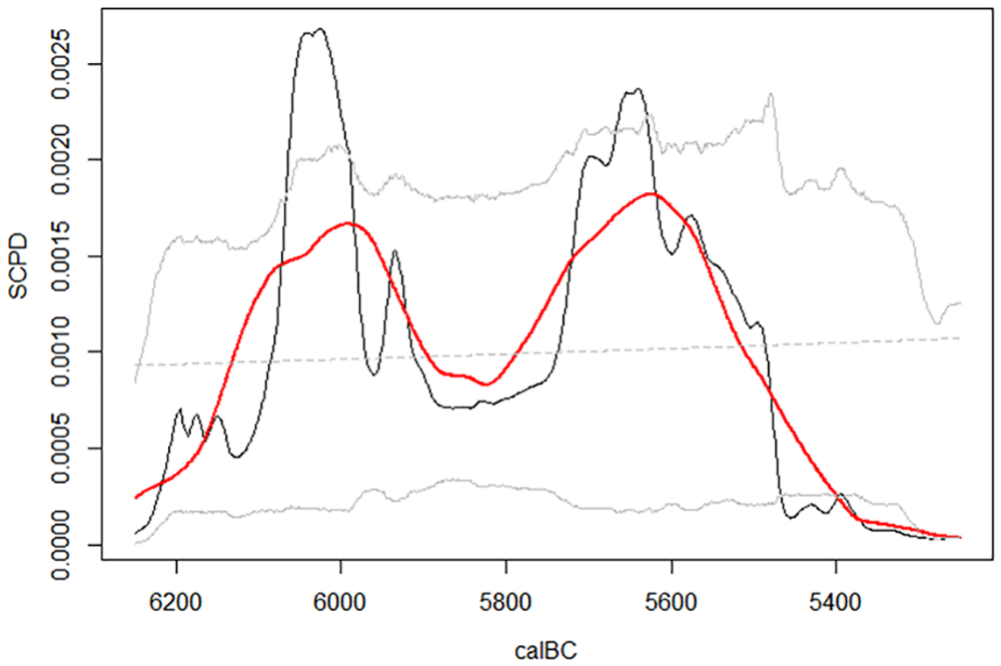
Results of the SCPD analysis based on the Early Neolithic radiocarbon dates from Serbia. SCPD empirical curve (black line) for Early Neolithic (Starčevo) dates, with 95% confidence intervals (gray lines), the null model (gray dashed line) and 200 year rolling mean (red line); number of dates = 72, number of bins = 21, global p value = .024.

The results of the SCPD method are shown in Fig 2. The empirical SCPD curve increases steeply between 6200 and 6000 calBC, beyond the upper 95% confidence interval limit. The statistically significant peak at ~6000 calBC is followed by a trough that reaches its lowest point between ~5900 and 5800 calBC, which is in turn followed by another statistically significant peak at ~5650 calBC. After this final peak, the SCPD curve plummets below the lower 95% confidence interval limit right after ~5500 calBC. This general trend is even clearer when we look at the 200 year rolling mean which smoothes the noise resulting from the calibration process. The global p value is 0.024 which indicates that the deviations from the null model are not likely to be false positives.

## Discussion

The significant peaks of the empirical SCPD curve should be interpreted as population increase while the significant troughs should be interpreted as population decrease relative to the null model which assumes that the population was stationary throughout the entire period and that the only factor which affected the frequency of radiocarbon dates from different parts of the period was loss due to taphonomy. The results indicate that there are three significant deviations from the null model which assumes stationary population during the Early Neolithic period: peak at ~6000 calBC, peak at ~5650, and trough after ~5500 calBC. It is also noteworthy that there is a pronounced trough in the SCPD curve between the first and the second significant peak. Although this trough is not statistically significant in itself, as the curve does not go below the lower 95% confidence interval limit, the fact that it is preceded by a statistically significant peak makes this trough also significant in the sense that the population must have decreased after the peak at least to the level preceding the first peak—when the SCPD curve goes back from outside to inside the 95% CIs it means that the curve is again consistent with the stationary population size before the significant deviation. Therefore we can deduce that, other things being equal, the trough between the two peaks corresponds to a population decrease, however a decrease which did not go below the value assumed by the null model.

The NDT theory implies that we should expect to find one significant peak soon after the introduction of the Neolithic in the area followed by a trough a few centuries later. However, we find two significant peaks with a trough between. If this population decrease was real then we would have a pattern which is different from what the NDT theory predicts and the patterns found in other regions of Europe by Shennan et al. [50] and Timpson et al. [51] as the initial population boom in Central Balkans would be followed by an immediate decrease with a rebound occurring 350 years later. This would mean that there was an abrupt increase in mortality or migration underlying the observed decrease, but the resulting population decrease was not catastrophic as the population size did not fall below the level predicted by the null model.

The alternative explanation for this pattern of a trough between two peaks is that it is a consequence of a research bias. The binning procedure within the Shennan-Timpson method controls for the research bias when it comes to differential dating of sites and site-phases for which the dated samples exist, but it does not account for the bias in the selection of sites from which the dates are sampled from in the first place. Given the importance of dating the earliest appearance of the Neolithic in the region, we would expect that researchers focused their sampling efforts to sites which were suspected to be the earliest in the sequence. The subdivision of Starčevo culture into phases was based on the changes in pottery decoration (for a review see [15, 76, 77]). However, the validity of these relative chronological schemes for the Early Neolithic of Central Balkans is questionable, as they are often contradictory, and it has been shown that they are weakly supported by absolute dates [15]. However it should be at least possible to discriminate between the earliest and latest Starčevo phases with more confidence [76], which would enable the researchers to intentionally choose the earliest sites and thus bias the results of the SCPD analysis in the manner seen in our results. An oversampling of the earliest sites would create such an artificial peak followed by a trough. If this was indeed the case, then the second peak in Fig 2 would correspond to the true NDT peak as observed in other regions of Europe.

Finally, there is a significant decrease of the SCPD curve after 5500 calBC which suggests that there was a substantial population decrease at the end of the Early Neolithic corresponding to the busts observed in other regional sequences in Europe. In order to control for possible edge effects, as the interval where the curve decreases is close to the limit of the analytical interval, we performed an additional analysis by adding the full set of published Late Neolithic (Vinča culture) radiocarbon dates (157 dates from 16 sites, S2 Database, Vinča sites) 2) from Serbia to the original set of Early Neolithic dates. It is important to emphasize that the Early and the Late Neolithic datasets are not strictly comparable due to different dating strategies in the two periods, so we had to apply corrections similar in principle to corrections made by Downey et al. [78] (the detailed description of the procedure for combining the Early and Late Neolithic dates into a single analysis is provided in electronic supplementary–S1 File Corrections for the joint Early and Late Neolithic curve sum). Due to corrections of the SCPD curve the validity of statistical tests would be questionable, so we only present the empirical SCPD curve, which should suffice to explore whether the trough after 5500 calBC will disappear if we include the Late Neolithic dates in order to remove the potential edge effect.

**Fig 3:**
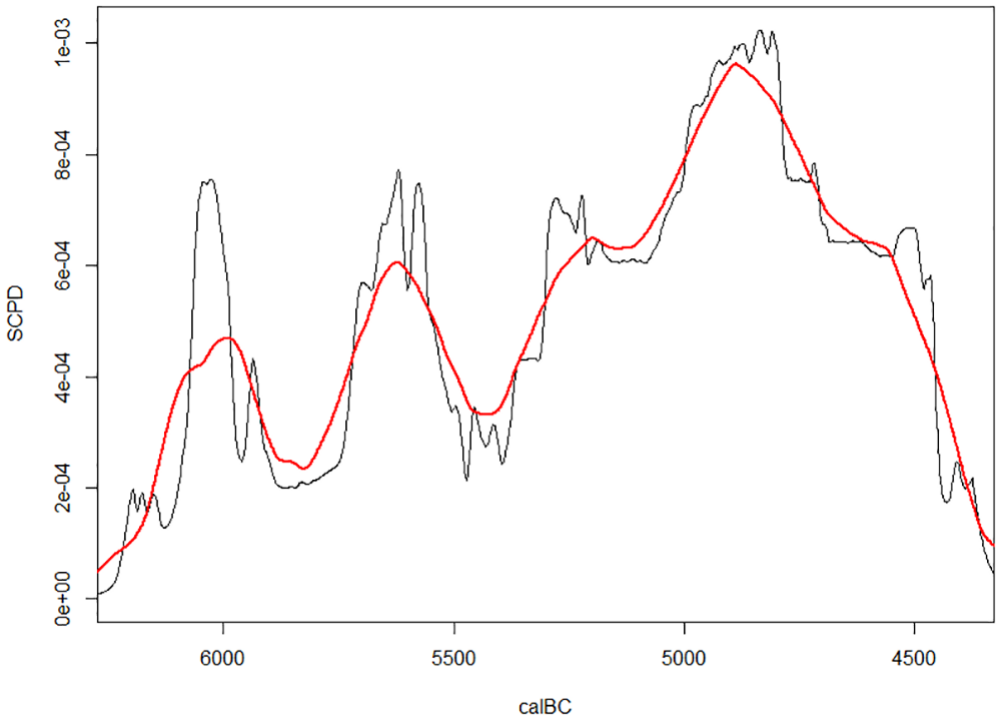
SCPD for the Early and Late Neolithic dates from Serbia. Empirical SCPD curve (black) for the sum of Early (Starčevo) and Late (Vinča) Neolithic dates with 200 year rolling mean (red).

As apparent from Fig 3 the trough persists, separating the Early and the Late Neolithic part of the population dynamics sequence. The demographic interpretation of this pattern would be that there was a population bust at the end of the Early Neolithic which marked the end of the Starčevo culture. The beggining of the Late Neolithic Vinča culture would coincide with the increase in population size, culminating in ~4800 calBC.

This population gap between the two cultures resonates well with the idea that demography is tightly linked with the formal variability of material culture in time and space, as cultural transmission of cultural traits, including the traits of the material culture, depend on population size and structure [56, 79]. The implication of this association between population and cultural transmission is that population bottlenecks would create drift-like patterns in the domain of material culture attributes (e.g. ceramic style) in a similar manner as in biological evolution. For example, the decrease in population size would also lead to the decrease of cultural diversity (e.g. diversity of pottery types). Variants that were rare before the bottleneck may become dominant simply by chance when the population starts to grow again after the bottleneck. The dynamics of this process are illustrated by means of computer simulation by Rorabaugh [80]. If the scenario of a substantial population decrease between Starčevo and Vinča culture is true, this might be an explanation for the differences in pottery styles between the Early and Late Neolithic ceramic assemblages. As many authors agree, Vinča or proto-Vinča elements (e.g. biconical bowls, figurine eye shapes) were present in Starčevo contexts at low frequencies [13, 81, 82], although the influx of new people by migration was also suggested as a part of the explanation of changes in material culture [83]. If the population bottleneck occurred, the change we observe in pottery styles between the Early and the Late Neolithic may have resulted from drift alone. The alternative to the bottleneck would be a population discontinuity between the Early and the Late Neolithic as the new population migrated from somewhere else into already depopulated Central Balkans ~5300 calBC.

It is also interesting to note that this pattern is also consistent with the recent empirical results by Manning et al. [84] who demonstrated that the SCPDs of radiocarbon dates grouped by traditionally defined archaeological cultures resemble Gaussian distributions—it seems as if each archaeological culture goes through the same three stages "gradual expansion—zenith—gradual disappearance" when the dates assigned to a particular culture are summed. The transition between Starčevo and Vinča seems to conform to this general pattern. Sites with mixed Starčevo and Vinča assemblages (with vessels produce with mixed technologies) may be particularly interesting in this regard [22, 85], although it is not likely that there was a bias against these sites in Serbian archaeology because the Serbian researchers artificially solved this problem by assuming two different phases on these sites purely on typological ground and in the absence of stratigraphic evidence [85]. Therefore, these sites would not stand out as culturally undetermined.

## Conclusion

The population dynamics of the Early Neolithic populations in Central Balkans in broadly consistent with the predictions of the NDT as there is clear evidence for population growth after the introduction of the Neolithic, and a strong indication of the population decline at the very end of the Neolithic period. The validity of the details of the pattern remains to be further investigated as it is not clear whether the population decrease right after 6000 calBC was real or the pattern is the artifact of the research bias towards dating the earliest Neolithic sites in region

The significance of these results is in the fact that they seem to suggest that cultural process in the Central Balkan Early Neolithic was similar to what is observed in the Early Neolithic of Central and Western Europe. The demographic dynamics produced by this process could have driven the spread of the Neolithic in a way consistent with predictions of the demic diffusion hypothesis.

## Supporting Information

S1 Database Starčevo sites with radiocarbon dates used in the study. (XLSX)

S2 Database Vinča sites with radiocarbon dates used in the study. (XLSX)

S1 File Corrections for the joint Early and Late Neolithic curve sum. (DOCX)
