# Supplementary material for: Demography of the Early Neolithic Population in Central Balkans: Population Dynamics Reconstruction Using Summed Radiocarbon Probability Distributions
Source: PLoS One. 2016 Aug 10;11(8):e0160832. doi: 10.1371/journal.pone.0160832 (PMC4980024; doi:10.1371/journal.pone.0160832)
Supplement: S1 File — (DOCX) [file pone.0160832.s003.docx]

# Correction made to the joint Early and Late Neolithic SCPD curve

Differences in site durations (e.g. differential residential mobility) can bias the results of the SPD method. If there are two equally sized populations, with differing site durations, one having 2 times higher site durations than the other, the number of sites coming from the population with shorter site durations would be two times higher and therefore the probability of finding and dating a sample from such site would be two times higher even though the population levels were equal. Differences in site size (area) can also bias the results. If there were two populations, where one population is twice the size of the other, but they live in the same number of settlements with the bigger population having bigger settlements, one date taken from a larger settlement would have the same weight as the date taken from a smaller settlement which would lead to a bias (given that we “know” that one population is bigger than another).

The number of known sites from the Late Neolithic (N = 514, count based on [1-2]) in Serbia is substantially higher than the number of known Early Neolithic sites (N = 333, count based on the database of Early Neolithic sites from Serbia collated for the purposes of the BEAN project) even though the Late Neolithic has somewhat shorter duration than the Early Neolithic. Likewise, the duration of EN sites was probably shorter than LN sites (which should result in more EN sites even if population size was constant between periods) and LN sites were on average several times bigger than EN sites. All this implies that the population in the LN was much higher than in the EN, but SCPD method would not be able to detect this because the number of LN dates is only slightly higher than the number of EN  dates and to make things worse they come from fewer sites than EN dates which further reduces the LN relative population size as they are more likely to be binned. For example, most of the dates from the Late Neolithic come from the large multilayered sites such as Vinča, Gomolava, Selevac, Belovode, Pločnik, while the number of dates per site is much smaller for the Early Neolithic even if there was no other source of bias, this fact alone would artificially inflate the values of the SCPD curve associated with the Early Neolithic as binning procedure would reduce the contribution of the Late Neolithic dates to the overall sum. For these reasons it was necessary to make a correction, similar in principle to the correction made by [3], in order to reduce these biases and get the more realistic population dynamics pattern.

The average thickness of deposits in the Late Neolithic was ~2 times higher than in the Early Neolithic, If the thickness of cultural deposits is taken as a rough proxy measure for the site temporal span, other things being equal, there would be more Early Neolithic than Late Neolithic sites even if the corresponding population levels were the same in both periods. Available data on site areas from the Late Neolithic in Serbia are in most cases unreliable [4], and for the Early Neolithic such data is practically non-existent (we found data only for two sites Blagotin and Jariciste, with areas in both cases being equal to 1ha, JasnaVuković and Miroslav Marić, personal communication), so we had to rely on the dataset on site areas from the same period (and culture) from Hungary [5]. The ratio of average Late to average Early Neolithic site area was 1.22. This is a very conservative correction given that the average area of the sites from which the dates actually come from in our samples in ~10ha so the actual difference is probably even greater.

These two ratios were used to make corrections of the SCPD curve in the following way. First, we summed the Early Neolithic dates according to the procedure described in the main text and normalized the area under the SCPD curve. Then we separately summed the Late Neolithic dates, normalized the area under the curve, and multiplied the curve values with 2.44 which is the product of the deposit ration and site area ratio (1.22*2). We then summed the Early and weighted Late Neolithic curves and normalized the sum. We did not perform the Monte Carlo simulation to calculate the confidence intervals as it is not clear what are the implications of this correction on the sample size and statistical power of the test.

This correction makes the Late Neolithic part of the curve higher than the Early Neolithic part of the curve as it should, but it does not influence the shape of the curve within the two periods. The correction only reduces the bias as it only takes into account differences in site size and duration, but does not account for the fact that the sampling strategies between periods were completely different and do not reflect true differences in the number of sites. Therefore, the resulting SCPD curve is certainly not realistic, it only roughly approximates the true population dynamics with the primary purpose of removing the edge effect at the end of the Early Neolithic SCPD curve.

# References

1. Chapman J. The Vinča Culture of South East Europe. Oxford: BAR; 1981.

2. Srejović D, editor. The Neolithic of Serbia. Belgrade: University of Belgrade, Faculty of Philosophy, Centre for Archaeological Research; 1988.

3. Downey SS, Bocaege E, Kerig T, Edinborough K, Shennan S. The neolithic demographic transition in Europe: correlation with juvenility index supports interpretation of the Summed Calibrated Radiocarbon Date Probability Distribution (SCDPD) as a valid demographic proxy. PLoS ONE. 2014;9(8).

4. Ristić-Opačić J. Topografsko-hronološke karakteristike naselja vinčanske kulture na teritoriji Srbije. Glasnik Srpskog arheološkog društva. 2005;21:71-112.

5. Anders A, Siklósi Z. The First Neolithic Sites in Central/South-East European Transect. In: Anders A, Siklósi Z, editors. The Koros Culture in Eastern Hungary: British Archaeological Reports; 2012.
